# The Role of Skeletal Muscle Mitochondria in Colorectal Cancer Related Cachexia: Friends or Foes?

**DOI:** 10.3390/ijms232314833

**Published:** 2022-11-27

**Authors:** Britt van de Haterd, Kenneth Verboven, Frank Vandenabeele, Anouk Agten

**Affiliations:** 1 REVAL—Rehabilitation Research Center, Faculty of Rehabilitation Sciences, Hasselt University, Agoralaan, 3590 Diepenbeek, Belgium; 2 BIOMED—Biomedical Research Center, Hasselt University, Agoralaan, 3590 Diepenbeek, Belgium; 3 U-RISE—Uhasselt Research Group on Innovative and Society-Engaged Education, School for Educational Studies, Hasselt University, Agoralaan, 3590 Diepenbeek, Belgium

**Keywords:** colorectal cancer, skeletal muscle, cachexia, mitochondria, inflammation

## Abstract

Up to 60% of colorectal cancer (CRC) patients develop cachexia. The presence of CRC related cachexia is associated with more adverse events during systemic therapy, leading to a high mortality rate. The main manifestation in CRC related cachexia is the loss of skeletal muscle mass, resulting from an imbalance between skeletal muscle protein synthesis and protein degradation. In CRC related cachexia, systemic inflammation, oxidative stress, and proteolytic systems lead to mitochondrial dysfunction, resulting in an imbalanced skeletal muscle metabolism. Mitochondria fulfill an important function in muscle maintenance. Thus, preservation of the skeletal muscle mitochondrial homeostasis may contribute to prevent the loss of muscle mass. However, it remains elusive whether mitochondria play a benign or malignant role in the development of cancer cachexia. This review summarizes current (mostly preclinical) evidence about the role of skeletal muscle mitochondria in the development of CRC related cachexia. Future human research is necessary to determine the physiological role of skeletal muscle mitochondria in the development of human CRC related cachexia.

## 1. Introduction

Cancer is associated with high morbidity and mortality, and it is an important public health problem [1]. Colorectal cancer (CRC) is the second leading cause of cancer related death in developed countries, the second most common cancer in women, and the third most common cancer in men [2,3]. Together, CRC comprises 11% of all cancer diagnoses and 5.8% of all cancer deaths. Usually, CRC emerges from the glandular, epithelial cells of the large intestine. Driving factors behind the development of CRC are obesity, sedentary lifestyle, red meat consumption, alcohol, and tobacco use [4]. The presence of cachexia is one of the underlying factors related to the high mortality rate of CRC, and is identified as a risk factor for adverse events during systemic therapies, thereby limiting treatment outcomes [5,6]. Cancer cachexia is a multifactorial syndrome characterized by involuntary weight and skeletal muscle mass loss, with or without loss of fat mass [7]. Depending on the type of cancer, the prevalence of cachexia goes up to 80%, with gastrointestinal and pulmonary cancers having the highest rates [8]. In advanced CRC, up to 60% of the patients develop cachexia. It is often diagnosed at a late stage when it coexists with excess body weight [9]. Cancer cachexia is strongly associated with chemotherapy induced toxicity, poor prognosis, and worse clinically relevant outcomes, such as fatigue, quality of life, and physical status [10,11,12,13]. Over the last years, overall survival of cachectic CRC patients has improved due to improvements in systemic therapy treatment. However, underlying mechanisms involved in the development of cancer related cachexia remain largely elusive. Therefore, fundamental research is necessary for further optimization of therapy and clinical care for cancer patients [10,11,12,13]. 

Skeletal muscle is one of the most abundant and most plastic tissues in the human body. It is the main protein reservoir in the body, accounting for approximately 40% of total body weight. Muscle mass depends on a balance between protein synthesis and protein degradation. Of interest, skeletal muscle wasting is the main manifestation of cancer cachexia [7]. It has been suggested that this results from an imbalance between skeletal muscle protein synthesis and degradation, with a net more protein degradation [14,15]. However, it is still unclear whether an increase in catabolic (protein degradation) or a decrease in anabolic (protein synthesis) processes (mutually) dominate in the development of cancer cachexia. Furthermore, this imbalance may depend on the duration of the disease [7]. An important aspect in gaining or preserving skeletal muscle mass and in improving muscle function in cancer patients is physical exercise [16,17,18]. 

Interestingly, mitochondria fulfill an established role in muscle atrophy. Due to their role in energy production, apoptotic processes, production of reactive oxygen species (ROS), and oxidation of muscle contractile proteins, these organelles are important regulators of skeletal muscle mass [19,20,21]. Evidence shows that systemic inflammation, oxidative stress, and proteolytic systems contribute to mitochondrial dysfunction in cancer cachexia, which (jointly) contribute to an imbalanced metabolism of skeletal muscle proteins [19,20,22]. Preclinical and in vivo mouse models for CRC related cachexia (C26 and *APC^Min/+^*) demonstrate a lower mitochondrial content, reductions in mitochondrial enzymatic activities involved in oxidative phosphorylation, and altered mitochondrial morphology and dynamics [19,20,21,23,24,25,26,27].The C26 mouse model is a well-characterized and extensively used mouse model for cancer cachexia. These mice bear the colon-26 tumor, also referred to as adenocarcinoma. This results in a 10% tumor weight versus total body weight and a reduction of 20–25% in skeletal muscle weight [28]. Another mouse model for CRC is the *APC^min/+^* model. These mice develop multiple colon adenomas and adenocarcinomas, and carry a heterologous mutation in the *Apc* gene, which is a tumor-suppressor gene in the Wnt signaling pathway [29]. They develop progressive cachexia between 12 and 20 weeks of age, with a decrease of 20–25% in body weight [30]. However, only a few studies investigated the role of skeletal muscle mitochondria in the development of CRC cachexia. The aim of this review is to summarize the existing literature about the role of skeletal muscle mitochondria in the development and progression of CRC related cachexia.

## 2. Underlying Mechanisms of Mitochondrial Dysfunction

In CRC related cachexia, systemic inflammation, oxidative stress and proteolytic systems contribute to the development of mitochondrial dysfunction, leading to skeletal muscle wasting [19,20,22]. Maintenance of the skeletal muscle mitochondrial homeostasis may be crucial to prevent skeletal muscle mass loss in cancer related cachexia [31].

### 2.1. The impact of Systemic Inflammation on Skeletal Muscle Mitochondria in CRC Cachexia

Systemic inflammation is a key driver in the development of cancer related cachexia by disrupting the balance between protein synthesis and protein degradation [32]. Pro-inflammatory factors that are released by cells during inflammation increase the production of ROS causing oxidative stress. This can either increase skeletal muscle protein degradation or decrease protein synthesis, and induce skeletal muscle mitochondrial dysfunction in CRC related cachexia [10,33,34,35,36]. Furthermore, these pro-inflammatory factors target several signaling pathways playing a possible role in the development of cancer related cachexia by causing mitochondrial dysfunction leading to muscle loss [37,38]. The contribution of inflammatory pathways in the development of CRC related cachexia has been studied in pre-clinical research using the C26 colon cancer and the *APC^min/+^* mouse model [19,21]. Pro-inflammatory mediators, such as IL-6, can activate these signaling pathways by binding specific receptors (IL-6 receptor-alfa) [39]. The most important and most investigated pathway within CRC cachexia and mitochondrial dysfunction is the Janus Kinase/signal transducers and activators of transcription 3(JAK/STAT3) pathway. Binding of IL-6 will lead to the activation of the JAK/STAT3 pathway [40,41]. Phosphorylation and activation of STAT3 will cause dimerization, nuclear translocation, DNA binding, and target gene regulation [40]. Eventually this will lead to tumor growth, dysregulation of mitochondrial respiration, biogenesis, fusion and fission, and muscle wasting [19,37,42]. Furthermore, mutations in components of the mitogen activated protein kinase/extracellular signal regulated kinase (MAPK/ERK) pathway will result in cells with malignant properties [43]. It has been shown that ERK inhibition prevents muscle wasting in C26 mice [44]. Additionally, activation of the phosphatidylinositol 3-kinase (PI3k)/Akt pathway by insulin growth factor 1 is downregulated in animal models of skeletal muscle atrophy [45,46,47]. This is caused by alterations in the PI3k/Akt effector molecules Foxo1/3, which are responsible for the expression of ubiquitin-ligases MAFbx and MuRF1. As such, the suppression of the PI3k/Akt pathway is linked to the activation of the ubiquitin-dependent proteolytic machinery, which is a hallmark of skeletal muscle wasting [46]. 

In cancer cachexia, the pro-inflammatory mediator IL-6 is associated with the dysregulation of skeletal muscle mitochondria [19,37,42]. In *APC^min/+^* mice, it was shown that there is no development of cachexia when they lack IL-6, while overexpression of IL-6 promoted cancer cachexia [19], the latter being associated with increased levels of phosphorylated STAT3 in skeletal muscle tissue [37]. The IL-6-STAT3 pathway plays a pivotal role in driving skeletal muscle wasting by driving skeletal muscle mitochondrial dysfunction. Skeletal muscle oxidative capacity is reduced in both oxidative and glycolytic skeletal muscles from *APC^min/+^* mice [19]. These effects on mitochondrial respiration in cachexia are important because oxidative phosphorylation (OXPHOS), coupling the electron transfer system to ADP (adenosine diphosphate) phosphorylation, can affect the redox status, oxidative stress levels, and thus mitochondrial dynamics and function. Eventually, this dysregulation of mitochondrial respiration could lead to protein degradation and skeletal muscle atrophy [48]. Evidence shows that there is cachexia-associated loss of muscle mitochondrial respiratory capacity in C26 mice [21,42,48,49]. Proteins involved in mitochondrial OXPHOS, including complex I (nicotinamide adenine dinucleotide hydrogen; NADH), complex II (succinate dehydrogenase; SDH), complex III (ubiquinol-cytochrome c reductase), complex IV (cytochrome c oxidase; COX), and complex V (ATP (adenosine triphosphate) synthase), are downregulated in skeletal muscle tissue from cachectic C26 mice [42,48]. These findings coincide with dysregulated nicotinamide adenine dinucleotide (NAD)^+^ metabolism and decreased muscle protein synthesis, occurring through the STAT3 pathway [42]. Furthermore, current results described in the literature suggest that the STAT3 pathway at least partly drives skeletal muscle wasting in a CRC mouse model (HCT116) since the expression of key proteins (AKT, ERK, P38) involved in other important signaling pathways were unaltered in this model [37].

### 2.2. Mitochondrial Biogenesis, Fusion and Fission in CRC Cachexia

An important regulator of mitochondrial biogenesis is peroxisome proliferator-activated receptor-gamma coactivator (PGC)-1α. This transcriptional coactivator is downregulated in cachectic skeletal muscle tissue, which was associated with a reduced oxidative capacity, further leading to muscle wasting [19,37,42]. NAD^+^ and sirtuin 1 (SIRT1), which both regulate mitochondrial oxidative metabolism, have PGC-1α as a downstream target. The levels of NAD^+^ and SIRT1 are significantly lower in untreated cachectic C26 mice. Treatment with soluble activin receptor (sACVR) replenishes NAD^+^ levels and normalizes SIRT1 expression similar to the predicted activities of PGC-1α [42]. Furthermore, antioxidant protection by restoring glutathione levels in skeletal muscle tissue of tumor-bearing mice is offered by sACVR treatment [50]. This possibly points out that sACVR can enhance cellular processes and mitochondrial function, indicating that PGC-1α and thus mitochondrial biogenesis is a key player in maintaining mitochondrial and skeletal muscle function [42]. Of interest, Ballaro et al., described that overexpression of PGC-1α in skeletal muscle of C26 mice was unable to prevent cancer or chemotherapy induced muscle mass loss, regardless of its ability to maintain mitochondrial oxidative capacity [27]. 

**Figure 1:**
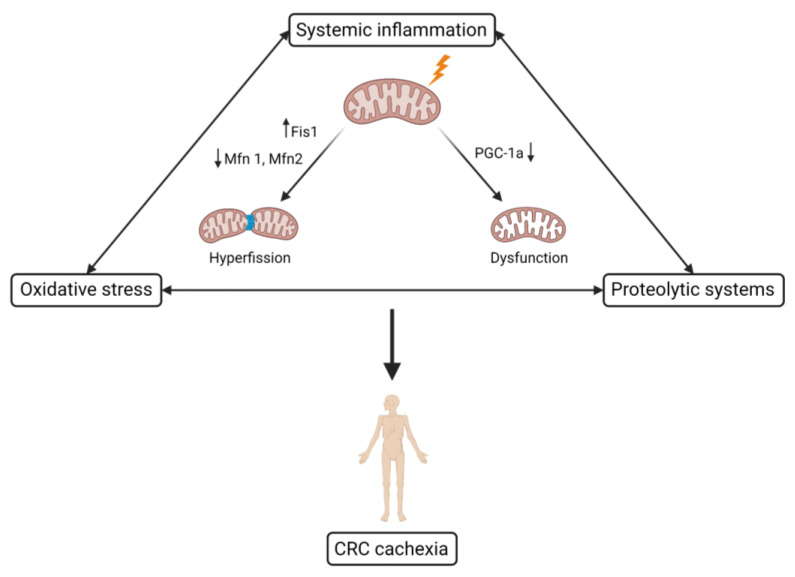
Schematic overview of the underlying mechanisms leading to skeletal muscle mitochondrial dysfunction in colorectal cancer related cachexia. Fis1, mitochondrial fission protein 1; Mfn1, mitofusin 1; Mfn2, mitofusin 2; PGC-1α, peroxisome proliferator-activated receptor-gamma coactivator 1α; CRC, colorectal cancer. Created with Biorender.com (accessed on 26 November 2022).

In order to adapt to different environmental and developmental contexts, mitochondria change their shape through fusion and fission. This is important for maintaining a physiologically healthy pool of mitochondria [51]. Mitochondrial fusion causes multiple mitochondria to fuse together, resulting in elongated mitochondria. On the other hand, mitochondrial fission will result in smaller mitochondria by splitting single mitochondria. Besides mitochondrial biogenesis, alterations in mitochondrial fusion and fission, caused by augmented STAT3 signaling also contribute to the development of CRC related cachexia [19,37,42]. Important regulators of mitochondrial fusion are Mfn1 and Mfn2. Knock out of these fusion regulators will result in muscle atrophy. Reductions in Mfn1 and Mfn2 expression are observed in *APC^min/+^* mice [19,37] and C26 mice [31]. Furthermore, in cachectic skeletal muscle tissue, the mitochondrial protein OPA1, which is also involved in mitochondrial fusion, is downregulated [37]. The loss of fusion proteins causes mitochondrial fragmentation, making them predisposed to apoptosis [19]. Additionally, Fis1, a regulator in mitochondrial fission, is upregulated in skeletal muscle tissue from cachectic *APC^min/+^* mice, leading to apoptosis and muscle mass loss [19]. Dynamin-related protein 1 (DRP1) is another pivotal factor of mitochondrial dynamics as inhibition of DRP1 has a negative effect on mitochondrial fission, with an appearance of elongated mitochondria. Overall, the loss of mitochondrial homeostasis caused by reduced mitochondrial biogenesis and fusion and more mitochondrial fission results in an increased ROS production. This will cause a reduction in muscle oxidative capacity and aggravated skeletal muscle atrophy in CRC related cachexia by promoting protein catabolic functions (Figure 1) [19,24,52].

Furthermore, reduced expression of MEF2C, which plays an important role in skeletal muscle development, is associated with changes in muscle structural integrity and mitochondrial function. Specifically, reduced MEF2C will lead to dysregulation of oxygen transport and ATP regeneration in skeletal muscle of C26 mice. Morphological changes in the mitochondria of cachectic skeletal muscle include loss of cristae and swollen mitochondria, suggesting defective oxidative phosphorylation [26].

### 2.3. The role of Proteolytic Systems in Skeletal Muscle Mitochondrial Dysfunction in CRC Cachexia

In skeletal muscle tissue, four main proteolytic systems orchestrate protein degradation (proteolysis) and mitochondrial dysfunction: 1) the macroautophagy system, (2) the ubiquitin-proteasome-dependent pathway (UPS), (3) the calpain system, and (4) the caspase pathway [53,54,55]. The macroautophagy system has an important function in the onset of skeletal muscle depletion in cancer cachexia by targeting skeletal muscle mitochondria. It is known that excessive autophagy has a negative effect on skeletal muscle function and impairs muscle mass. In C26 mice, autophagic bodies are observed within skeletal muscle mitochondria, suggesting mitochondrial loss by autophagy (mitophagy) and dysfunction of muscle energy homeostasis [26]. However, partial blockade of autophagy does not ameliorate tissue wasting in C26 mice, which might indicate that autophagy is only partially responsible for skeletal muscle wasting in CRC related cachexia, being accompanied by other proteolytic systems, such as calpains and the proteasome [22].

Of interest, survival of C26 mice is not negatively affected when autophagy is induced. However, muscle protein wasting is exacerbated when excessive autophagy together with increased UPS activity cause degradation of structural or functional (mitochondrial) proteins [22]. 

Zeng et al. showed that activation of mitochondrial calpain induces mitochondrial injury and cell damage. Coculture of myoblasts with colon carcinoma cells activates calpains in myotube mitochondria causing non-selective pore opening on the inner membrane of mitochondria (MPTP) and mitochondrial membrane potential (Δψ_m_) alterations, together resulting in mitochondrial injury. Furthermore, mitochondrial respiration becomes altered by an impaired OXPHOS complex I activity in myotube mitochondria [56]. Additionally, they showed that inhibition of calpain improves the function of OXPHOS complex I and thus mitochondrial respiration [56]. This could implicate that there is upregulated activation of the calpain system in CRC related cachexia mouse models, contributing to muscle atrophy. 

## 3. Skeletal Muscle Mitochondrial Disruption Leads to Apoptosis in CRC Cachexia

**Figure 2:**
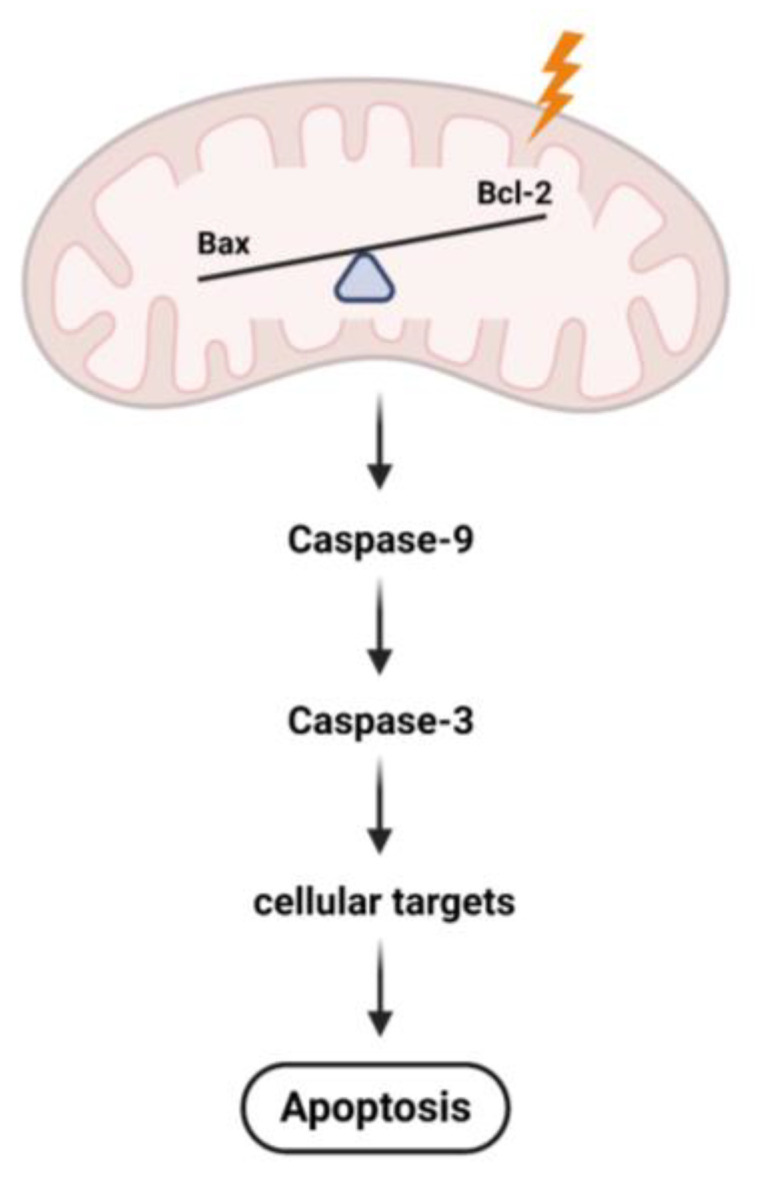
Disrupted Bax-to-Bcl-2 ratio in mitochondria of C2C12 myoblasts coculture with CT26 colon carcinoma cells. Bcl-2, B-cell lymphoma 2; Bax, Bcl-2-associated X protein. Created with Biorender.com (accessed on 11 October 2022).

The most common mechanism of myocyte apoptosis is a mitochondrial-centered control pathway. Here, changes in Δψ_m_ serve as a marker for mitochondrial function. Apoptotic signals converge at mitochondrial membranes causing the loss of Δψ_m_, leading to the release of toxic proteins into the cytosol [57]. These toxic proteins form apoptosomes, which will trigger the caspase pathway, leading to the activation of the downstream pathway involved in apoptotic cellular dismantling and clearance [57]. Coculture of C2C12 myoblasts with CT26 colon carcinoma cells increases the Bax/Bcl-2 ratio, leading to activation of the caspase pathway in mitochondria, and eventually apoptosis and muscle atrophy (Figure 2) [57,58,59]. Zeng et al. showed that adding either acylated ghrelin (AG) or unacylated ghrelin (UnAG) to the cocultures prevented the loss of Δψ_m_. Ghrelin is a multifunctional circulating hormone that exists in two different forms (AG and UnAG). The receptors of ghrelin are widely expressed in skeletal muscle tissue and play important roles in immune function and muscle oxidative metabolism in both humans and animals [60,61]. Specifically, both AG and UnAG inhibited the activation of caspase-3 and thereby protects myoblasts from apoptosis by inhibiting mitochondrial dysfunction induced by CT26 colon carcinoma cells. AG and UnAG activate Akt (increased p-Akt/Akt ratio) and ameliorate the decreased levels of Bcl-2 in mitochondria. Thereby, both AG and UnAG suppress myoblast apoptosis [57]. These findings suggest that both AG and UnAG can be possibly used in the treatment of cancer cachexia. Furthermore, Miao et al. showed that exosomes secreted by the C26 mouse cells decreased the diameter of C2C12 myotubes together with a decrease in muscle strength. Results showed that inhibition of exosome secretion ameliorated muscle wasting in C26 mice. Certain miRNAs (miR-195a-5p and miR-125b-1-3p) were richer in C26 mice exosomes compared to non-cachectic derived exosomes. It was shown that these miRNAs activated the apoptotic signaling, also by downregulating Bcl-2, and thereby triggering the caspase pathway in skeletal muscle mitochondria [58]. Additionally, Zhang et al. recently showed that cachectic C26 mice derived exosomes are rich in growth differentiation factor 15, which induces muscle atrophy of cultured C2C12 myotubes by regulating the Bcl-2/caspase-3 pathway [59]. 

## 4. The Effect of Exercise on Skeletal Muscle Mitochondrial Function in CRC Cachexia

In healthy persons [62] and cancer patients [63,64], physical exercise is associated with better health outcomes and health related quality of life. In mice, physical exercise increases total mitochondrial protein content within skeletal muscle fibers and thereby activates AMPK, the upstream regulator of PGC-1α [65,66]. However, the effects of exercise on skeletal muscle mass loss has been limitedly investigated in C26 mice. Here, different types of exercise (resistance, endurance, low intensity, high intensity) have been studied [31,67,68].

In C26 mice performing endurance training only (voluntary wheel running), OXPHOS subunit proteins and mitochondrial PGC-1α become upregulated. Moreover, exercise normalizes markers of oxidative stress and prevents abnormal mitochondrial morphology in skeletal muscle tissue of C26 mice. Interestingly, endurance trained C26 mice showed an increased food intake, a better grip strength, and showed a negative effect on tumor growth [31]. The combination of endurance and resistance training showed similar results as endurance training only [31,67]. The combined exercise training showed a trend towards more PGC-1α, cytochrome C, and SDH expression in skeletal muscle tissue of C26 mice. Hence, both endurance as well as combined training positively affects muscle mass and function by improving mitochondrial function [67].

Exercise, by using motorized wheel running, increased skeletal muscle mass and strength in C26 mice [27,68]. It caused a reduction of ROS levels, thereby decreasing oxidative stress and restoration of redox homeostasis in the skeletal muscles of exercised C26 mice [68]. Furthermore, motorized wheel running led to increased mitochondrial biogenesis and function (PGC-1α, cytochrome C, and SDH), and was able to partially reduce the expression of mitophagy markers (BNIP3) [27,68]. Additionally, exercised C26 mice show increased levels of Mfn2 mRNA, but no differences in the expression of Mfn1 [27], suggesting that exercise had a positive effect on mitochondrial fusion in these mice. 

Exercise can also be combined with erythropoietin (EPO) to investigate the effects on muscle alterations in cancer cachexia. The receptor from EPO is located in the skeletal muscle and promotes myoblast differentiation and survival by the activation of MAPK and Akt [69]. The combination of EPO administration and exercise in C26 mice prevents partially cross-sectional area (CSA) reduction and prevents a shift from oxidative to glycolytic fiber type. Furthermore, acute exercise for two weeks combined with EPO has an anti-inflammatory effect, by reducing circulating levels of the pro-inflammatory cytokine IL-6. However, on a long term, this effect was lost, which could be explained by the fact that prolonged exercise also releases IL-6. The combination of exercise and EPO rescues skeletal muscle mitochondrial function and structure in C26 mice, suggesting that EPO has a fundamental role in mitochondrial function. However, the administration of EPO alone in C26 mice is unable to prevent accumulation of dysfunctional mitochondria, indicating that exercise plays an important role [70]. 

## 5. Conclusions and Future Perspectives

This review describes current knowledge about the role of skeletal muscle mitochondria in CRC related cachexia. However, skeletal muscle mitochondrial dysfunction can also be observed in other cancer types besides CRC [24,71,72]. Based on the existing literature, it is undeniable that deterioration of skeletal muscle mitochondria plays a pivotal role in the development of CRC related cachexia. Several preclinical studies showed altered mitochondrial oxidative capacity, biogenesis, and fusion and fission, in CRC related cachexia. Therefore, preserving and/or restoring mitochondrial quality could be a promising future therapeutic strategy to maintain or improve muscle function and muscle mass in CRC patients. However, further research in human studies is absolutely necessary to unravel the role of skeletal muscle mitochondria in human CRC related cachexia. Of particular interest, (preventive) exercise could be a promising intervention to improve mitochondrial function, ultimately aiming to prevent or improve CRC related cachexia.

## Data Availability

Not applicable.
